# SIPL1, Regulated by MAZ, Promotes Tumor Progression and Predicts Poor Survival in Human Triple-Negative Breast Cancer

**DOI:** 10.3389/fonc.2021.766790

**Published:** 2021-12-17

**Authors:** Juanjuan He, Jing Wang, Teng Li, Kunlun Chen, Songchao Li, Shaojin Zhang

**Affiliations:** ^1^ Department of Breast Surgery, The First Affiliated Hospital, Zhengzhou University, Zhengzhou, China; ^2^ Department of Urology Surgery, The First Affiliated Hospital, Zhengzhou University, Zhengzhou, China; ^3^ Department of Hepatobiliary and Pancreatic Surgery, The First Affiliated Hospital, Zhengzhou University, Zhengzhou, China

**Keywords:** MAZ, SIPL1, triple-negative breast cancer, Akt, NF-κB

## Abstract

**Background:**

Triple-negative breast cancer (TNBC) is an aggressive subtype of breast cancer owing to a lack of effective targeted therapy and acquired chemoresistance. Here, we explored the function and mechanism of shank-interacting protein-like 1 (SIPL1) in TNBC progression.

**Methods:**

SIPL1 expression was examined in human TNBC tissues and cell lines by quantitative reverse transcription PCR, western blot, and immunohistochemistry. *SIPL1* overexpression and silenced cell lines were established in BT-549 and MDA-MB-231 cells. The biological functions of SIPL1 in TNBC were studied *in vitro* using the CCK-8 assay, CellTiter-Glo Luminescent Cell Viability assay, caspase-3/8/9 assay, wound healing assay, and transwell assay and *in vivo* using a nude mouse model. The potential mechanisms underlying the effects of SIPL1 on TNBC progression were explored using bioinformatics analysis, luciferase reporter assays, and chromatin immunoprecipitation followed by qPCR.

**Results:**

*SIPL1* expression was higher in human TNBC tissues and cell lines than in adjacent normal tissues and a breast epithelial cell line (MCF10A). High expression of *SIPL1* was positively correlated with poor overall and disease-free survival in patients with TNBC. *SIPL1* overexpression elevated and *SIPL1* silencing repressed the malignant phenotypes of TNBC cells *in vitro*. *SIPL1* overexpression promoted xenograft tumor growth *in vivo*. Myc-associated zinc-finger protein (MAZ) transcriptionally activated *SIPL1*. Finally, we found that SIPL1 promoted TNBC malignant phenotypes *via* activation of the AKT/NF-κB signaling pathways.

**Conclusions:**

These results indicate that the MAZ/SIPL1/AKT/NF-κB axis plays a crucial role in promoting the malignant phenotypes of TNBC cells.

## Introduction

Breast cancer (BC) is the most commonly diagnosed malignancy in women, accounting for 10% of new malignancies diagnosed worldwide (1). Triple-negative breast cancer (TNBC), a subtype of breast cancer with the absence of estrogen receptor (ER), progesterone receptor (PR), and human epidermal growth factor receptor 2 (HER2), represents approximately 15–20% of all breast cancers (2). Although a few targeted therapies are currently available for TNBC, chemotherapy remains the critical approach for TNBC treatment (3, 4). Thus, it is imperative to investigate novel molecular targets for TNBC diagnosis and treatment.

Shank-interacting protein-like 1 (SIPL1), also known as Shank-associated RH domain-interacting protein (SHARPIN), is a component of the linear ubiquitin assembly complex (LUBAC) (5). Although quite a few studies have shown that *SIPL1* is amplified and overexpressed in many types of human malignancies, such as breast cancer, gastric cancer, and melanoma (6–8), some studies have demonstrated the tumor-suppressive role of *SIPL1* in esophageal cancer progression (9). Zhou et al. *demonstrated* that *SIPL1* expression was elevated in human melanoma tissues and SIPL1 overexpression promoted melanoma development and progression *via* the p38 and c-Jun N-terminal kinases (JNK)/c-Jun signaling pathways (7). Melo et al. reported that SIPL1 may repress the expression and function of phosphatase and tensin homolog deleted on chromosome 10 (PTEN) in CHO-K1 cells, which promotes tumorigenesis by activating phosphoinositide 3-kinase (PI3K)/AKT signaling (10). In breast cancer, *SIPL1* expression is associated with tamoxifen resistance (6, 11) and may increase ERα protein levels by augmenting its monoubiquitination (12). In addition, *SIPL1* knockdown decreases the number and size of metastases in nude mouse xenograft tumors (13). In view of the wide-ranging roles of *SIPL1* proposed in breast cancer, a comprehensive summary of the mechanisms by which *SIPL1* regulates TNBC tumorigenesis has not been directly reported.

The Myc-associated zinc-finger protein (*MAZ*) gene, located on chromosome 16p11.2, was recently identified as a transcription factor that drives inflammation in animal models (14). Several studies suggest that *MAZ* expression is dysregulated in many types of human cancers, such as BC, colorectal cancer, gastric cancer, and prostate carcinoma (15–18). These findings indicate that MAZ promotes proliferation, epithelial–mesenchymal transition (EMT), and cancer invasion by activating multiple downstream target genes at the transcriptional level.

In the present study, we examined the biological functions of SIPL1 in human TNBC cells. Our results show that SIPL1 promotes cell viability, apoptosis resistance, and cell migration in TNBC cell lines. Furthermore, SIPL1 promotes xenograft tumor growth in orthotopic mammary fat pad models *in vivo*. Our results further demonstrated that MAZ transcriptionally activated *SIPL1*, which further activated the AKT/NF-κB signaling pathways. Our data will be of great significance for the prevention and therapy of TNBC.

## Materials and Methods

### Cell Culture and Transfection

Human TNBC cell lines (MDA-MB-231, MDA-MB-436, MDA-MB-468, and BT-549) were purchased from the American Tissue Culture Collection (ATCC, Manassas, VA, USA). BT-549 cells were cultured in RPMI-1640 medium (Corning, NY, USA) with 10% (v/v) fetal bovine serum (FBS; Hyclone US origin, GE Lifescience) and 1% penicillin/streptomycin (Gibco-BRL). Other cell lines were maintained in Dulbecco’s Modified Eagle Medium (DMEM; Gibco, Grand Island, NY, USA) plus 10% FBS and 1% penicillin/streptomycin. MCF10A, a breast epithelial cell line, was cultured in the DMEM/F12 medium (Gibco) containing 5% horse serum, cholera enterotoxin (0.1 μg/ml), insulin (10 μg/ml), l-glutamine (2 mM), epidermal growth factor (20 ng/ml), and hydrocortisone (0.5 μg/ml). All cell lines were maintained in a humidified atmosphere with 5% CO_2_ at 37 °C. Regular mycoplasma testing was conducted using a PCR-based assay.

Short hairpin RNA (shRNA) against *SIPL1* (shSIPL1#1, shSIPL1#2) or *MAZ* (shMAZ) as well as shRNA negative control (shNC) were synthesized by Genepharma (Shanghai, China; **Supplementary Table S1**). The full-length complementary DNA (cDNA) of human *SIPL1* or *MAZ* was synthesized and cloned into the expression vector pcDNA3.1. When they reached 60–70% confluence, the MDA-MB-231 and BT-549 cells were transiently transfected with the corresponding vector. Cells were collected 24 h after transfection for subsequent analyses. Transfection of vectors was performed using Lipofectamine 3000 reagent (Invitrogen, Carlsbad, California, USA) following the manufacturer’s instructions. For inhibitor studies, LY294002—an inhibitor of PI3K—(1.0 μM, Sigma Aldrich) and Bay 11-7082—an inhibitor of IKKα—(5.0 μM, Sigma Aldrich) were dissolved in dimethyl sulfoxide (DMSO). An equivalent volume of DMSO was used as vehicle control.

For *in vivo* experiments, lentiviral vectors carrying *SIPL1* (pLenti-U6-SIPL1-GFP, Lv-SIPL1) and purified negative control particles (pLenti-U6-NC-GFP, Lv-NC) were designed and synthesized by Genepharma (Shanghai, China). BT-549 cells (4 × 10^5^ per well) were transduced with these lentiviral vectors (1 × 10^8^ transducing units per ml), including an antibiotic resistance gene against puromycin, using polybrene (5 µg/ml). Cells were treated with 5 μg/ml puromycin to select stable cells. Transduction efficiency was estimated using fluorescence microscopy (Olympus IX71; Olympus Corporation) on the basis of the percentage of GFP-positive cells, quantitative reverse transcription PCR (qRT-PCR), and western blotting. The sequence was confirmed by DNA sequencing.

### RNA Isolation and Quantitative Reverse Transcription PCR

Total RNA was isolated using the TRIzol reagent (Invitrogen) according to the manufacturer’s protocol. Reverse transcription of extracted RNA (1 µg) was performed using MMLV Superscript reverse transcriptase and random hexamers, which yielded a final concentration of 50 ng/µl cDNA. The cycle conditions were as follows: 50 °C for 2 min, then 95 °C for 10 min, followed by 40 amplification cycles (95 °C for 15 s, 60 °C for 60 s). Gene expression relative to the expression of the housekeeping gene glyceraldehyde-3-phosphate dehydrogenase (GAPDH) was calculated using the 2^−ΔΔCT^ method (19). The primers are listed in **Supplementary Table S1**.

### Western Blotting

RIPA buffer (Thermo Scientific) containing Complete EDTA-free protease inhibitor was used to isolate proteins from cells and tissues. Proteins (30 μg) were loaded with a 4–15% TGX gel (BioRad) and then transferred to 0.2 µm PVDF membranes (BioRad). The membranes were blocked in 5% non-fat dried milk/Tris-buffered saline and 0.1% Tween-20 for 1 h at room temperature. Next, the membranes were incubated with primary antibodies: anti-SIPL1 (Abcam, ab79039), anti-MAZ (Abcam, ab85725), anti-AKT (Abcam, ab18785), anti-p-AKT (Abcam, ab38449), anti-P65 (Abcam, ab32536) or anti-p-P65 (Abcam, ab76302), and anti-GAPDH (Abcam, ab9485) overnight, along with horseradish peroxidase-labeled IgG (Abcam, ab205718) at room temperature. The protein bands were detected using enhanced chemiluminescence (Pierce, Rockford, IL, USA). Densitometry analysis was performed using ImageJ software (version 1.36; National Institutes of Health, Bethesda, MD, USA).

### Cell Proliferation

We used the CCK-8 assay (Beyotime Institute of Biotechnology, Shanghai, China) and CellTiter-Glo Luminescent Cell Viability assay to examine cell proliferation following the manufacturer’s instructions. For the CCK-8 assay, TNBC cell lines (1.0 × 10^3^ per well) were transiently transfected with shRNAs or overexpression plasmids and cultured for 24 h. The absorbance at 570 nm in each well was measured at 0, 1, 2, 3, and 4 d by an enzyme-linked immunosorbent assay (ELISA) plate reader. For CellTiter-Glo Luminescent Cell Viability assay, TNBC cells (1.0 × 10^3^ per well in 96-well plates) were cultured overnight at 37°C. Then, 100 µl CellTiter-Glo solution (Promega Corporation, Madison, WI, USA) was directly mixed with the culture medium and incubated at room temperature for 20 min. Following this, the luminescence intensity was recorded. Experiments were repeated at least three times.

### Caspase Activity

Cell apoptosis was investigated using the Caspase-3/8/9 colorimetric assay kits (Abcam, USA) following the manufacturer’s instructions. In brief, the TNBC cells (MDA-MB-231 and BT-549) were collected and lysed in ice-cold cell lysis buffer and then seeded in 24-well plates and incubated at 37°C for 12 h. A microtiter plate reader (Benchmark, Bio-Rad, USA) was used to assess the samples.

### Wound Healing Assay

TNBC cells were seeded in a 12-well plate and grown to 100% confluency. Using a p20 pipette tip, a scratch was made in each cell monolayer. After washing with phosphate-buffered saline (PBS) three times, the cells were cultured in complete medium for 24 h. Images were taken at 0 h and 24 h after wounding using a Leica optical microscope. Wound closure rate at 24 h was expressed as percentage of the original wound area.

### Transwell Assay

Cell migration and invasion were examined using transwell chambers with 8 μm pore size (BD Biosciences, USA) pre-coated without (for migration) or with (for invasion) Matrigel (BD Biosciences). TNBC cell lines (5 × 10^4^) were incubated in an FBS-free medium and were seeded in the upper chamber with Matrigel-coated or uncoated membrane. Medium plus 10% FBS as an attractant was added to the lower chamber. The cells were allowed to migrate for 24 h. The migratory and invaded cells on the lower surface were stained with 0.1% crystal violet solution. Three independent fields for each insert were photographed under a microscope (Olympus, Tokyo, Japan).

### Animal Experiments

All animal experiments were approved by the Ethics Committee for Animal Experiments of Zhengzhou University (approval no. 20170213). Female BALB/c nude mice (6 weeks of age) were obtained from Shanghai Laboratory Animal Center (Shanghai, China). All mice were kept and fed under specific pathogen-free conditions. MDA-MB-231 and BT-549 cells (5 × 10^6^) stably expressing *SIPL1* or control vector in 50% Matrigel (Corning, #354248) were injected into the bilateral mammary fat pads of female BALB/c nude mice. Body weight and tumor growth were monitored every 5 days. Tumor volume was measured using calipers and calculated using the following formula: 0.52 × length × width^2^. Five weeks following the inoculation, tumor samples were excised, weighed, and harvested for further examination.

### Human Sample Collection

In total, 119 women diagnosed with TNBC were recruited from the Department of Breast Surgery, the First Affiliated Hospital, Zhengzhou University (He’nan, China) between January 2011 and May 2012. The clinicopathological characteristics of these 119 patients with TNBC are shown in **Supplementary Table S2**. We acquired all patients’ consent from the Institutional Research Ethics Committee to use the clinical specimens for research. In addition, 24 archived fresh TNBC specimens were also obtained from the First Affiliated Hospital of Zhengzhou University. The patients received no adjuvant anti-cancer therapy before surgery. All patients were staged according to the 2010 American Joint Committee on Cancer (AJCC) tumor–node–metastasis (TNM) classification (20). The histological type was defined using the World Health Organization’s classification system (21).

### Immunohistochemistry

For immunohistochemical (IHC) assessment of SIPL1 and Ki-67 in breast tumor tissues from the nude mice and patients with TNBC, the DAKO Envision system (DAKO, Carpinteria, California) was used following a previously described method (22). In brief, the sections were blocked in 3% hydrogen peroxide, and they were incubated with primary antibodies to SIPL1 or Ki-67. The staining results were scored by the degree of staining intensity (0, full negative; 1, weak [light yellow]; 2, moderate [yellow-brown]; 3, robust [brown]) and the percentage of positive staining cells (0, full negative; 1, <10%; 2, 10–35%; 3, 35–75%; 4, >75% positive cells). Thus, the final scores were used to classify the specimens into two groups: score <6 defined as low expression and score 7–12 defined as high expression. The results of the IHC staining were evaluated by two pathologists blinded to the clinic pathological data.

### Luciferase Reporter Assay

TNBC cells (4 × 10^4^ cells per well) were plated in 12-well plates for 24 h. Then, cells were co-transfected with SIPL1 promoter-reporter plasmids (*PGL3*/*SIPL1*), *MAZ* overexpression plasmid, or pcDNA3.1, in addition to 5 ng pRL-TK Renilla plasmid (Promega, Madison, WI) using Lipofectamine 3000 (Invitrogen). After 36 h, both Luciferase and Renilla signals were detected by a dual-luciferase reporter gene detection kit (Promega, Madison, WI) according to the manufacturer’s instructions.

### Chromatin Immunoprecipitation

In a 10 cm dish, 1% formaldehyde solution was added to TNBC cells (1.5 × 10^7^) for 10 min at room temperature. Then, the cells were washed three times with PBS and harvested in chromatin immunoprecipitation (ChIP) lysis buffer supplemented with protease inhibitor. After the cells were lysed for 20 min, cells were sonicated on ice to shear the DNA into 0.1–0.5 kb fragments. The supernatant was centrifuged at 12,000 rpm for 15 min. Immunoprecipitation was then performed by incubating with anti-MAZ antibody (Abcam, ab85725) overnight at 4°C. Then, 60 μl of protein G agarose was added to the DNA–protein complexes overnight, and the associated genomic DNA was amplified by real-time PCR.

### Bioinformatic Analysis

To compare *SIPL1* expression between primary tumors and nontumor tissues, we extracted *SIPL1* expression values from Ualcan (http://ualcan.path.uab.edu/). The correlation analysis of *SIPL1* and *MAZ* expression was performed using BRCA datasets from GEPIA (http://gepia.cancer-pku.cn). The potential upstream transcriptional factors that induced high *SIPL1* expression were predicted using the online tool PROMO (23) and GeneCards (https://www.genecards.org/). MAZ binding sites on the *SIPL1* promoter were predicted using the JASPAR database (http://jaspar.genereg.net/).

### Statistical Analysis

Each experiment was repeated three times, and all values were presented as mean ± SD. All analyses were executed using SPSS 24.0 (SPSS Inc., Chicago, IL, USA) and GraphPad Prism version 8 (GraphPad Inc., La Jolla, CA, USA). Student’s t-test, analysis of variance (ANOVA), Spearman correlation test, and chi-squared test were used as appropriate. Survival analyses were performed using the Kaplan–Meier method and Cox’s proportional hazards regression model. All tests were two tailed. A *P* value less than 0.05 was considered significant.

## Results

### Expression and Clinical Significance of SIPL1 in TNBC

**Figure 1:**
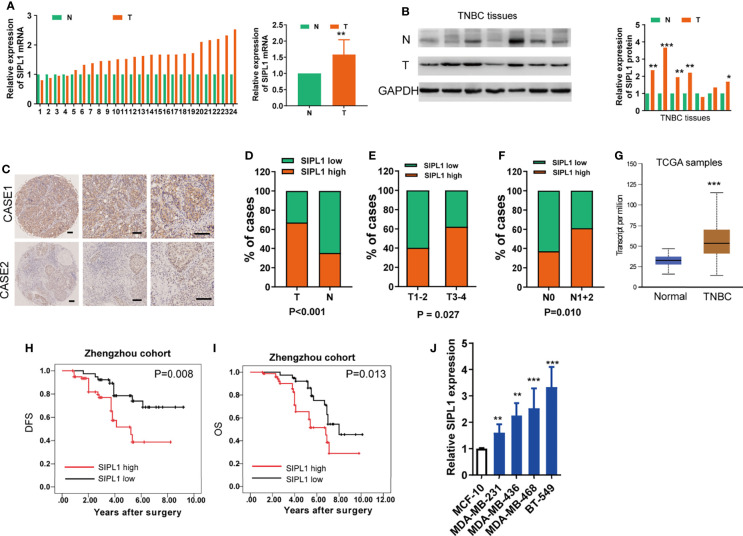
SIPL1 expression and clinical significance in human TNBC tissue samples and cell lines. **(A, B)** RT-qPCR and western blot assay analysis of SIPL1 expression in human tumor (T) and adjacent normal (N) breast tissues in patients with TNBC (n=24). Data were obtained using the 2^-ΔΔCT^ method and were normalized to GAPDH levels. **(C, D)** Representative immunohistochemical staining and histograms for SIPL1 expression were shown and compared between T and N breast tissues in patients with TNBC from the Zhengzhou cohort (n = 119). Scale bar, 100μm. **(E, F)**, High expression of SIPL1 was significantly associated with aggressive T stage **(E)** and node invasion **(F)** in patients with TNBC from the Zhengzhou cohort. **(G)** High expression of SIPL1 was significantly associated with tumor status according to TCGA-BRCA data set. **(H, I)** Kaplan-Meier survival curves showed that high expression of SIPL1 was related to dismal DFS and OS in patients with TNBC from the Zhengzhou cohort. **(J)** RT-Qpcr assay analysis of SIPL1 expression in TNBC cell lines. Data are presented as the mean ± SD from triplicate experiments. *P < 0.05; **P < 0.01; ***P < 0.001. TNBC, triple-negative breast cancer; T, tumor tissues; N, adjacent non-cancerous tissues; TCGA, the cancer genome atlas; BRCA, breast cancer; OS, overall survival; DFS, disease-free survival.

To determine the expression levels of *SIPL1* in TNBC, we performed qRT-PCR and western blotting assays in human TNBC (T) and corresponding non-tumor (N) tissues. Results showed that *SIPL1* mRNA and protein expression levels were significantly higher in tumor tissues than in adjacent non-cancer tissues (**Figures 1A, B**). Using a tissue microarray (TMA) of human TNBC tissues (n = 119) and IHC staining, we analyzed the clinical significance of *SIPL1* expression. By dividing the specimens into two groups based on the IHC score of *SIPL1* level, we showed that tumor tissues had higher expression levels of *SIPL1* than adjacent non-tumor tissues (**Figures 1C, D**). Furthermore, high expression levels of *SIPL1* were significantly correlated with tumor size (**Figure 1E**) and lymph node invasion (**Figure 1F**) but not with lymphovascular invasion and tumor grade (data not shown). We also explored *SIPL1* expression in the cancer genomic atlas (TCGA)-breast carcinoma (BRCA) datasheet, and the results were consistent with our findings (**Figure 1G**). Survival analyses showed that high levels of *SIPL1* were markedly associated with poorer overall survival (OS) and disease-free survival (DFS) in our cohort (**Figures 1H, I**). Univariate and multivariate Cox regression analysis confirmed high *SIPL1* expression as a predictor of low DFS (hazards ratio [HR] = 2.13, 95% CI: 1.17–3.87) and OS (HR = 2.88, 95% CI: 1.25–6.63; **Table 1**) in patients with TNBC. Additionally, the *SIPL1* expression levels in five TNBC cell lines were higher than those in MCF-10A, a breast epithelial cell line (**Figure 1J**).

**Table 1:** Univariate and multivariate Cox regression analyses of clinicopathologic characteristics for TNBC patients.

Clinical and pathologic Indexes	Univariate analysis	P	Multivariate analysis	P
**Overall survival**				
T stage, T3+T4 *vs*. T1+T2	2.18 (1.06-4.48)	0.035	2.45 (1.05-5.71)	0.038
Node involvement, yes *vs*. no	2.18 (1.03-4.60)	0.041	1.61 (1.00-2.58)	0.048
Grade: G3 *vs*. G1+G2	2.15 (0.91-5.07)	0.080	1.68 (0.96-2.94)	0.070
Lymphovascular invasion, yes *vs*. no	1.09 (0.47-2.49)	0.845		
Adjuvant chemotherapy, no *vs*. yes	1.73 (1.20-2.48)	0.003	1.54 (1.11-2.13)	0.009
Adjuvant radiotherapy, no *vs*. yes	0.95 (0.48-1.86)	0.874		
SIPL1 staining, high *vs*. low	2.33 (1.17-4.64)	0.017	2.88 (1.25-6.63)	0.013
**Disease-free survival**				
T stage, T3+T4 *vs*. T1+T2	1.69 (1.07-2.67)	0.025	1.56 (0.85-7.69)	0.094
Node involvement, yes *vs*. no	1.62 (1.19-2.20)	0.002	1.71 (1.08-2.70)	0.021
Grade: G3 *vs*. G1+G2	2.22 (1.38-3.60)	0.001	1.93 (1.22-3.05)	0.005
Lymphovascular invasion, yes *vs*. no	1.48 (0.66-3.33)	0.344		
Adjuvant chemotherapy, no *vs*. yes	1.74 (1.05-2.88)	0.031	1.59 (1.04-2.44)	0.033
Adjuvant radiotherapy, no *vs*. yes	1.16 (0.52-2.20)	0.875		
SIPL1 staining, high *vs*. low	2.21 (1.19-4.10)	0.012	2.13 (1.17-3.87)	0.013

### SIPL1 Overexpression Enhances Cell Proliferation, Apoptosis Resistance, and Migration and Invasion Abilities of TNBC Cell Lines *In Vitro*

**Figure 2:**
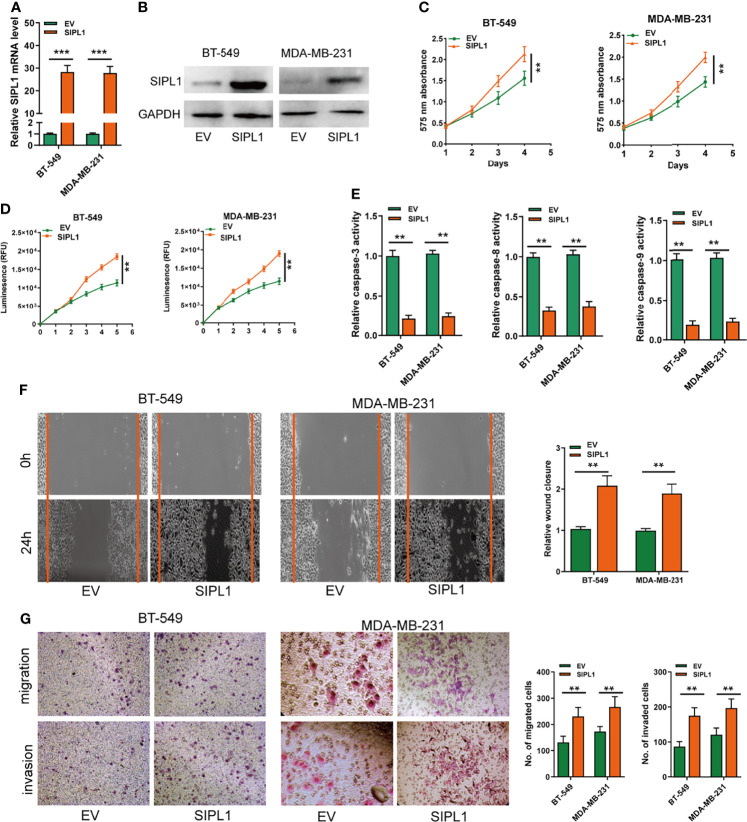
Overexpression of SIPL1 promotes cell proliferation, apoptosis resistance, migration and invasion in TNBC cell lines. **(A, B)** RT-qPCR and western blot assay analysis of the expression of SIPL1 in BT-549 and MDA-MB-231 cell lines transduced with SIPL1 vector compared to EV. Expression levels were normalized by GAPDH expression. **(C, D)**, CCK-8 **(C)** and CellTiter-Glo Luminescent Cell Viability Assay **(D)** analysis of the proliferative ability of BT-549 and MDA-MB-231 cells transduced with the indicated vectors. **(E)** The activity of caspase-3, -8, and -9 assay analysis of cell apoptosis ofBT-549 and MDA-MB-231 cells transduced with the indicated vectors. **(F)** Wound healing assay analysis of cell migration in BT-549 and MDA-MB-231 cell lines transduced with the indicated vectors. **(G)** Transwell assay without or with matrigel analysis of cell migration and invasion in BT-549 and MDA-MB-231 cell lines transduced with the indicated vectors. Error bars represent the mean ± standard deviation of three independent experiments. A t-test was used to evaluate the statistical significance as compared to the control. **P < 0.01; ***P < 0.001. TNBC, triple-negative breast cancer.

To explore the effects of *SIPL1* on the progression of TNBC cell lines *in vitro*, BT-549 and MDA-MB-231 cell lines were transduced with the *SIPL1* overexpression vector (SIPL1) and empty vector (EV). The data showed that *SIPL1* levels were evidently augmented in the two TNBC cell lines, as evidenced by qRT-PCR and western blotting (**Figures 2A, B**). Afterward, CCK-8 and CellTiter-Glo Luminescent Cell Viability assays showed that cell viability of cells transduced with ectopic SIPL1-expressing vectors was higher than that of cells transduced with control vectors (**Figures 2C, D**). Furthermore, caspase-3/8/9 assays showed that *SIPL1* overexpression significantly repressed TNBC cell apoptosis (**Figure 2E**). Wound healing and transwell assays showed that the migration and invasion abilities were augmented in cells with *SIPL1* overexpression (**Figures 2F, G**). These data indicate that *SIPL1* overexpression boosts malignant phenotypes of TNBC cells.

### SIPL1 Knockdown Hampers Cell Proliferation, Apoptosis Resistance, and Migration and Invasion Abilities of TNBC Cell Lines *In Vitro*

**Figure 3:**
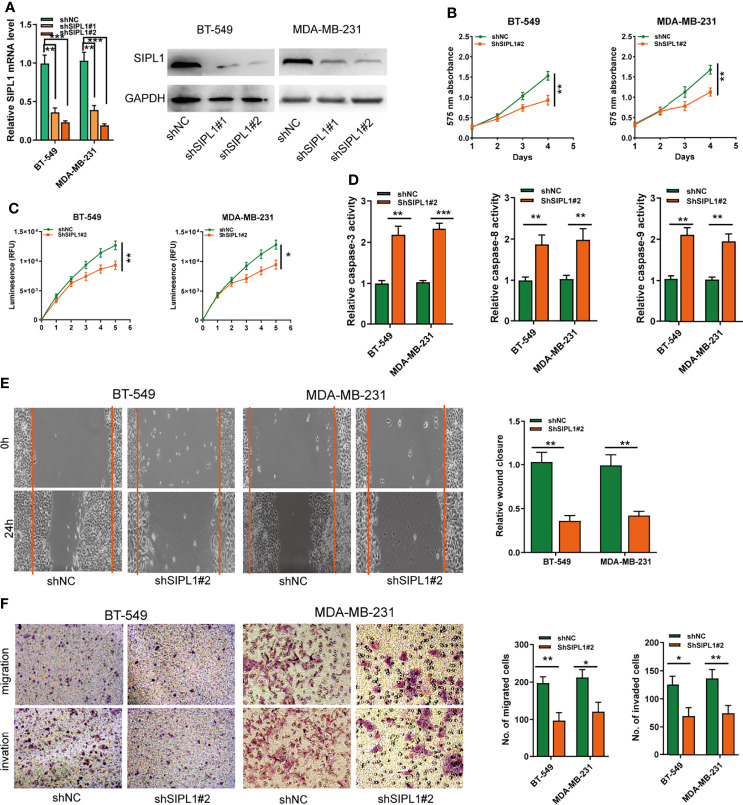
Knockdown of SIPL1 inhibits cell proliferation, apoptosis resistance, migration, and invasion in TNBC cell lines. **(A, B)** RT-qPCR and western blot assay analysis of the levels of SIPL1 in BT-549 and MDA-MB-231 cell lines transfected with two parallel shRNA targeting SIPL1 or shNC. **(C, D)**, CCK-8 **(C)** and CellTiter-Glo Luminescent Cell Viability Assay **(D)** analysis of the proliferative ability of BT-549 and MDA-MB-231 cells transfected with shSIPL1#2 or shNC. **(E)** The activity of caspase-3, -8, and -9 assay analysis of cell apoptosis of BT-549 and MDA-MB-231 cells transfected with shSIPL1#2 or shNC. **(F)** Wound healing assay analysis of cell migration of BT-549 and MDA-MB-231 cells transfected with shSIPL1#2 or shNC. **(F, G)**, Transwell assay without **(F)** or with **(G)** matrigel analysis of cell migration and invasion ofBT-549 and MDA-MB-231 cell lines transfected with shSIPL1#2 or shNC. Error bars represent the mean ± SD of three independent experiments. *P < 0.05; **P < 0.01; ***P < 0.001. TNBC, triple-negative breast cancer; NC, negative control.

The effects of *SIPL1* silencing on the biological activities of TNBC cells were explored using two parallel shRNAs targeting SIPL1 (shSIPL1#1 and shSIPL1#2) for the knockdown experiments. qRT-PCR and western blotting assay validated the knockdown efficiency of both shRNAs in the two cell lines, but shSIPL1#2 showed a higher efficiency (**Figure 3A**). The CCK-8 and CellTiter-Glo Luminescent Cell Viability assay demonstrated a marked decrease in cell proliferation in BT-549 and MDA-MB-231 cell lines transfected with shRNAs targeting *SIPL1* (**Figures 3B, C**). Furthermore, results of the caspase-3/8/9, wound healing, and transwell assays indicated that *SIPL1* knockdown significantly prohibited the cell apoptosis resistance, migration, and invasive ability of BT-549 and MDA-MB-231 cells (**Figures 3D–F**). These data suggest that *SIPL1* knockdown inhibited TNBC cell malignant phenotypes *in vitro*.

### SIPL1 Promotes TNBC Tumor Growth in Nude Mice *In Vivo*

**Figure 4:**
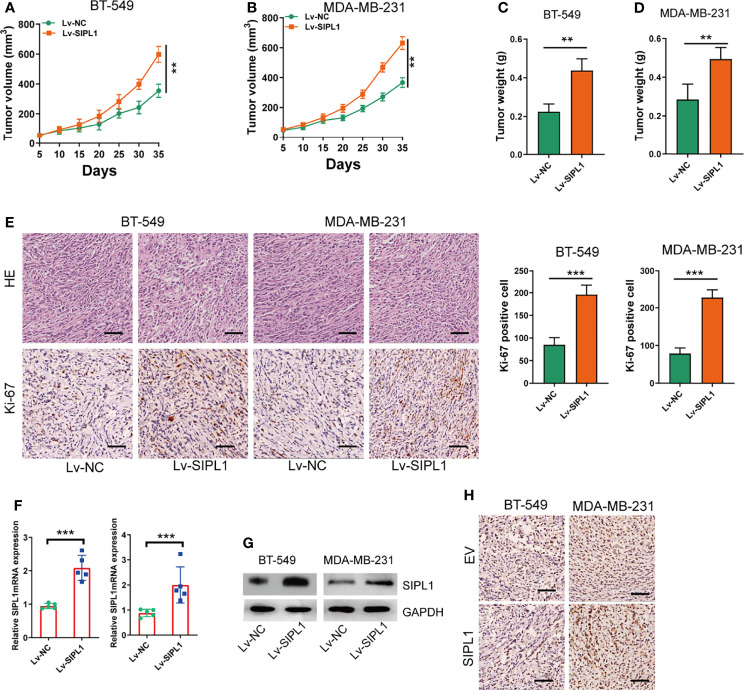
SIPL1 promotes tumor growth of TNBC *in vivo.* **(A, B)** Measurement of the volumes of xenograft tumors in the four indicated groups of mice. Scale bar, 1.0cm. **(C, D)** Measurement of the weights of xenograft tumors in the four indicated groups. **(E)** Representative H&E and Ki-67-stained sections in xenograft tumor in the four indicated groups of mice. Scale bar, 200μm. **(F–H)** RT-qPCR, western blot, and immunohistochemical staining analysis of SIPL1 expression in xenograft tumors in the four indicated groups of mice. Error bars represent the mean ± standard deviation of three independent experiments. **P < 0.01; ***P < 0.001. Lv, lentivirus; NC, negative control.

To determine the effects of *SIPL1* on TNBC growth *in vivo*, BT-549 and MDA-MB-231 cells (1 × 10^7^) were transduced with stable overexpression vectors and control vectors and then injected subcutaneously into nude mice, respectively. Tumor volume (**Figures 4A, B**) and weight (**Figures 4C, D**) were higher in the *SIPL1* overexpression groups than in control groups. Similarly, SIPL1 overexpression enhanced tumor proliferation, as determined by Ki-67 expression levels (**Figure 4E**). Furthermore, qRT-PCR, western blotting, and IHC assay confirmed the higher levels of *SIPL1* expression in the *SIPL1* overexpression groups than in control groups (**Figures 4F–H**). These data suggest that *SIPL1* contributes to TNBC tumorigenesis *in vivo*.

### MAZ Transcriptionally Activates SIPL1

**Figure 5:**
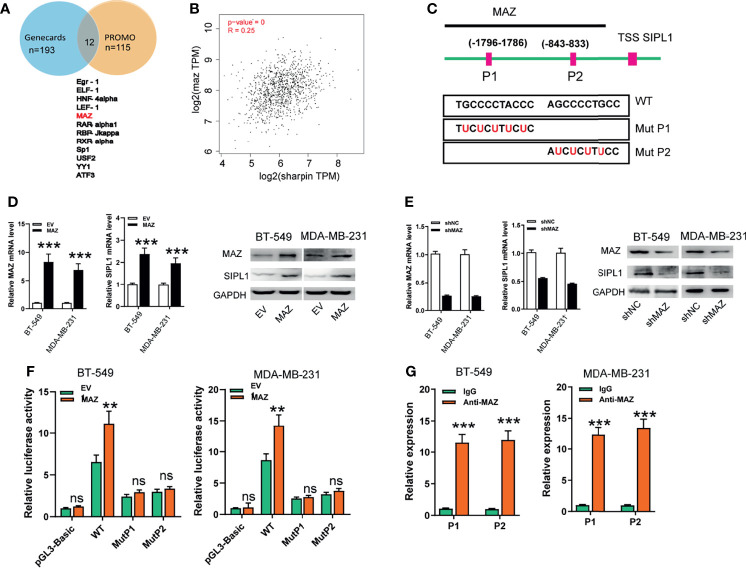
MAZ transcriptionally activates SIPL1 expression. **(A)** Using the online tool PROMO and gene cards, 11 transcriptional factors were shown. **(B)** MAZ was positively associated with SIPL1 expression in TCGA-BRCA dataset. **(C)** Using the JASPER bioinformatics software program, two MAZ-binding motifs were identified, named P1 and P2. **(D, E)** RT-qPCR and western blot assay analysis of MAZ and SIPL1 expression in BT-549 and MDA-MB-231 cells transduced with MAZ or shMAZ vector and control vectors. Data were obtained using the 2^-ΔΔCT^ method and were normalized to GAPDH levels. **(F)** Luciferase reporters assay analysis of luciferase activities of BT-549 and MDA-MB-231 cells transduced with MAZ vector and WT or mut SIPL1 promoter. **(G)** ChIP followed by qPCR assays analysis of MAZ expression in BT-549 and MDA-MB-231cells. Error bars represent the mean ± standard deviation of three independent experiments. **P < 0.01; ***P < 0.001.WT, wide type; mut, mutant type; TNBC, triple-negative breast cancer; TCGA, the cancer genome atlas; TSS, transcription start site. ns,not significant.

To determine the specific upstream factors that induced high *SIPL1* expression in TNBC, we further examined the potential transcriptional factors (TFs) using the online tool PROMO and GeneCards and identified 11 TFs (**Figure 5A**). Among them, MAZ was focused on because it was positively associated with *SIPL1* expression in the TCGA-BRCA dataset (**Figure 5B**). Furthermore, using the JASPER online program, we searched a 2 kb region upstream of the transcription start site of SIPL1, and two MAZ-binding motifs from −1796 to −1786 and −843 to −833 were identified, named P1 and P2, respectively (**Figure 5C**). After successfully constructing the *MAZ* overexpression and silenced BT-549 and MDA-MB-231 cell lines, we confirmed that ectopic expression of *MAZ* increased and *MAZ* knockdown decreased *SIPL1* expression in TNBC cells (**Figures 5D, E**). Moreover, *MAZ* overexpression enhanced luciferase activity in the wild-type *SIPL1* promoter, and these effects were not observed when the P1 or P2 site was mutated (**Figure 5F**). To evaluate whether MAZ can bind to the two potential sites within the promoter of *SIPL1*, we conducted ChIP followed by qPCR in BT-549 and MDA-MB-231 cells with MAZ overexpression using an anti-MAZ antibody. The ChIP-qPCR assays showed enrichment within the two *SIPL1* promoter sites (**Figure 5G**). Overall, these results suggest that MAZ mediated the upregulation of *SIPL1* in TNBC cells by binding to the *SIPL1* promoter and increasing its transcription.

### MAZ Is Essential for the Malignant Phenotypes Promoted by SIPL1 in TNBC Cell Lines *In Vitro*

**Figure 6:**
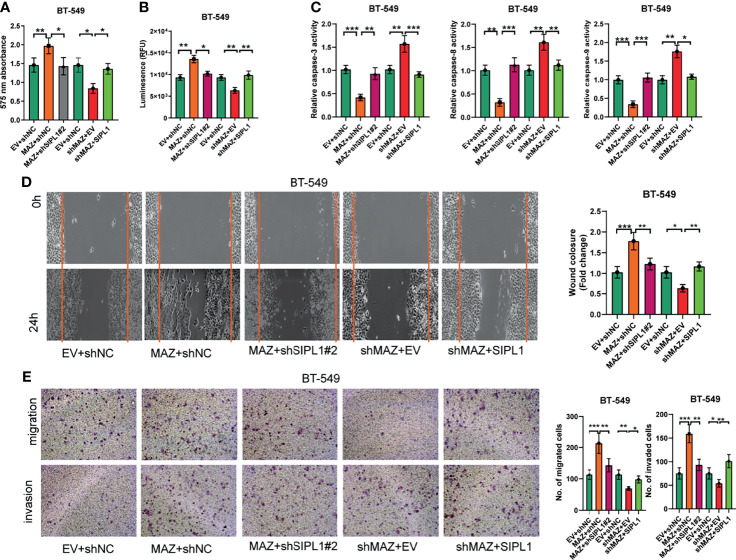
MAZ is essential for the tumorigenesis of SIPL1 *in vitro.* **(A–E)** CCK-8 **(A)**, CellTiter-Glo Luminescent Cell Viability **(B)**, the activity of caspase-3/8/9 **(C)**, wound healing **(D)**, and transwell assay [without or with matrigel, **(E)**] were performed in BT-549 cells transfected with the indicated vectors or negative control. Error bars represent the mean ± standard deviation of three independent experiments.*P < 0.05; **P < 0.01; ***P < 0.001. WT, wide type; TNBC, triple-negative breast cancer.

As demonstrated above, MAZ upregulates *SIPL1* expression at the transcriptional level in TNBC cells. Next, we hypothesized that MAZ is involved in the pro-tumorigenic role of SIPL1 *in vitro*. To this end, we constructed BT-549 cells with *MAZ* overexpression and endogenous SIPL1 silencing. We also constructed *MAZ* silencing with SIPL1 overexpression cell lines by knockdown of *MAZ* in *SIPL1*-overexpressing BT-549 cells. *MAZ* overexpression upregulated *SIPL1* expression, which was inhibited by shRNAs targeting *SIPL1*. Similarly, *SIPL1* expression was inhibited by *MAZ* knockdown but was upregulated by *SIPL1* overexpression (**Supplementry Figures 1A, B**). The additional functional experiment confirmed that *MAZ* overexpression significantly increased TNBC cell proliferation, apoptosis resistance, migration, and invasive abilities, whereas *SIPL1* silencing reversed these effects. In contrast, *MAZ* silencing led to reduced malignant phenotypes in TNBC cells, which was abrogated by *SIPL1* overexpression (**Figures 6A–E**). These findings indicate that *MAZ* mediated the pro-tumorigenic role of *SIPL1* in TNBC cell lines.

### SIPL1 Facilitated TNBC Tumorigenesis *via* Activation of the AKT/NF-κB Signaling Pathways

**Figure 7:**
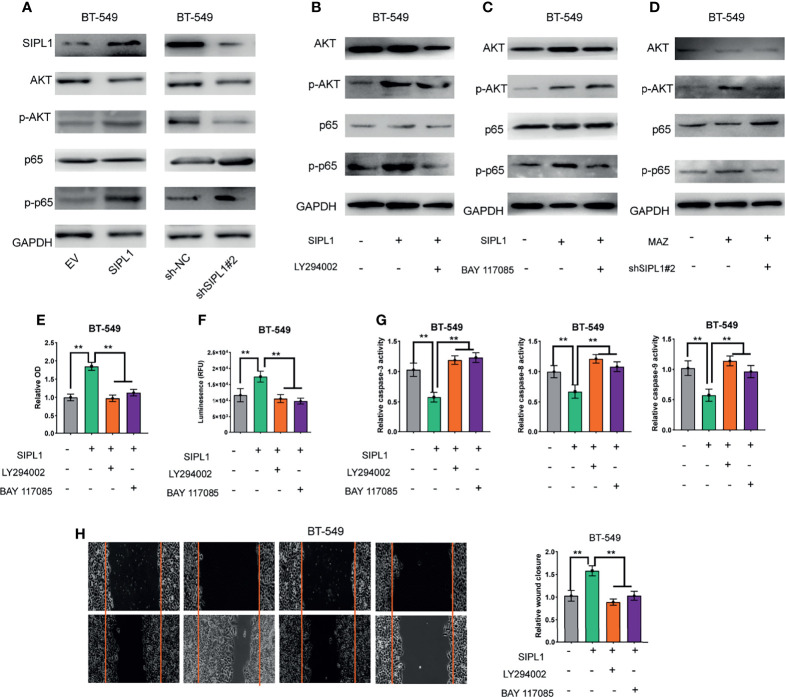
SIPL1 facilitates TNBC tumor progression via activation of AKT/NF-κB signaling pathways. **(A)** Western blot assay analysis of the activation of AKT and NF-*κB* signaling pathways in BT-549 cells transduced with SIPL1 or shSIPL1 plasmid and control vector. **(B)** Western blot assay analysis of the activation of AKT and NF-κB signaling pathways in BT-549 cells with SIPL1 overexpression and treatment of LY294002. **(C)** Western blot assay analysis of the activation of AKT and NF-κB signaling pathways in BT-549 cells with SIPL1 overexpression and treatment of BAY117085. **(D)** Western blot assay analysis of the activation of AKT and NF-κB signaling pathways in BT-549 cells with MAZ overexpression and knockdown of SIPL1. E-H, CCK-8 **(E)**, CellTiter-Glo Luminescent Cell Viability **(F)**, the activity of caspase-3/8/9 **(G)**, wound healing **(H)** assay analysis of cell viability, apoptosis resistance and migration in BT-549 cells with SIPL1 overexpression and treatment of LY294002 or BAY117085. **P < 0.01. TNBC, triple-negative breast cancer.

Previous studies have demonstrated that SIPL1 activates the PI3K/AKT and NF-κB signaling pathways in several human cancers (5, 24–26). Here, we examined whether both pathways participate in SIPL1-mediated TNBC tumor progression. As shown in **Figure 7A**, ectopic expression of *SIPL1* in BT-549 cells significantly promoted AKT and NF-κB, whereas *SIPL1* silencing led to the opposite effects. Moreover, LY294002 (a PI3K inhibitor) inhibited the SIPL1-mediated activation of the AKT/NF-κB signaling pathways (**Figure 7B**). BAY117085 (an NF-κB inhibitor) could inhibit the activation of the NF-κB signaling pathway but did not affect the activation of the AKT signaling pathway, indicating that SIPL1-mediated activation of the NF-κB signaling pathway is modulated by the PI3K/AKT pathway (**Figure 7C**). We further examined the effects of MAZ on both signaling pathways. Data showed that *MAZ* overexpression led to activation of the AKT and NF-κB pathways in BT-549 cells and the further knockdown of *SIPL1* deactivated both pathways (**Figure 7D**). Then, we examined whether both pathways participate in SIPL1-mediated cellular functions. The results revealed that the two inhibitors (LY294002 and BAY117085) restrained SIPL1-mediated BT-549 cell viability, apoptosis resistance, and migration (**Figures 7E–H**). Collectively, these data suggest that the AKT/NF-κB signaling pathways participate in SIPL1-mediated TNBC tumor progression.

## Discussion

We report here that *SIPL1* is significantly upregulated in TNBC tissues and cell lines, and high *SIPL1* expression is correlated with unfavorable clinicopathological characteristics and low survival in patients with TNBC. Moreover, *in vitro* experiments have confirmed that *SIPL1* overexpression augments the proliferation, apoptosis resistance, migration, and invasion abilities of TNBC cells, but *SIPL1* silencing restraints these biological functions in TNBC cells. Ectopic expression of *SIPL1* facilitated xenograft tumor growth *in vivo*. We also confirmed that *SIPL1* was upregulated by MAZ at the transcriptional levels and was involved in activating the PI3K/AKT and NF-κB signaling pathways.

SIPL1 was first identified to be necessary for intact immune response (26). Further studies revealed that SIPL1 was amplified and upregulated in many types of cancer and plays an essential function in cancer progression. For example, Zhang et al. confirmed that SIPL1 was upregulated in prostate cancer, and it contributed to cancer progression by enhancing the expression of survivin and livin (27). In addition, SIPL1 augments prostate cancer progression by modulating the expression of the apoptosis-associated protein (25). In breast cancer, *SIPL1* mRNA and protein expression is significantly upregulated, and the high expression is associated with lower disease-free survival (DFS) and OS (28). A previous study showed that injecting MDA-MB-231 cells with shRNAs targeting *SIPL1* into nude mice through the tail vein led to a significant reduction in the number and size of metastases (13). In the present study, using human TNBC tissue samples and IHC staining, we demonstrated that *SIPL1* mRNA and protein expression were upregulated. High *SIPL1* expression is correlated with advanced T stage, lymph node invasion, and low DFS and OS in patients with TNBC. TNBC differs from other human breast cancer subtypes, as it is highly proliferative and biologically more aggressive and exhibits a high likelihood of recurrence, distant metastasis, and mortality (29). Furthermore, *in vitro* functional experiments showed that *SIPL1* functions as an oncogene in human TNBC by augmenting cell mobility, apoptosis resistance, and invasion. We also generated two stable *SIPL1* overexpression TNBC cell lines and demonstrated that *SIPL1* overexpression promoted TNBC tumor growth *in vivo* in xenograft nude mice models. Collectively, our investigations confirmed the oncogene function of *SIPL1* in TNBC.

We explored the upstream mechanisms by which *SIPL1* contributes to TNBC progression and identified the involvement of the transcriptional factor MAZ. On the basis of ChIP and dual-luciferase reporter assay, our data confirmed the direct binding sites of MAZ and the *SIPL1* promoter; the binding eventually leads to upregulated expression of *SIPL1*. The functional rescue experiments indicated that MAZ is involved in the malignant phenotypes promoted by SIPL1 in TNBC cell lines *in vitro*. Notably, as SIPL1 may be directly and indirectly associated with gene regulatory networks in addition to MAZ, the possibility that SIPL1-associated biological roles may also be modulated independently by MAZ cannot be excluded. Recently, it has been reported that expression of *MAZ* is elevated in many types of human cancer, such as colorectal cancer, gastric cancer, breast cancer, and prostate carcinoma (15–18). These data indicate that MAZ facilitates cancer proliferation, EMT, and metastasis by activating multiple downstream target genes at the transcriptional level.

The NF-κB signaling pathway comprises a family of five transcription factor subunits (p65/RelA, c-Rel, RelB, p50/NF-κB1, and p52/NF-κB2), which are key regulators of tumor cell growth, proliferation, metastasis, and chemotherapy resistance (30, 31). Aberrant activation of the NF-κB pathway is frequently observed in TNBC, and inhibition of NF-κB activity can suppress the growth of TNBC cells (30, 32). Kuo et al. reported that treatment with *NF-κB* responsive element-driven suicide gene prohibited proliferation and invasion of TNBC cells (33). Additionally, some studies have shown that SIPL1 serves an essential role in cancer through modulating NF-KB and PTEN signaling (5, 24). Therefore, we investigate the downstream mechanisms mediating the SIPL1-associated biological functions. Our data showed that *SIPL1* overexpression in BT-549 cells activated AKT and NF-κB signaling by increasing AKT and p65 phosphorylation in BT-549 cells, whereas the inhibitors of two pathways inhibited the SIPL1-mediated activation of the AKT/NF-κB signal pathways. However, how SIPL1 regulates PI3K/AKT and NF-κB activity through the phosphorylation of AKT and p65 remains unclear. Recent studies have demonstrated that SIPL1, as a component of LUBAC, mediates linear ubiquitylation of endogenous NF-κB essential modulator (NEMO) and thus augments the activation of the NF-κB signaling pathway (34). In addition, recent reports have revealed that SIPL1 activates the AKT pathway by directly binding to and inactivating PTEN in human cancer cells both *in vitro* and *in vivo* (24). Loss of PTEN is the primary mechanism that activates the PI3K/AKT pathway in mice and cells (35, 36).

Taken together, the present study has shown that *SIPL1* expression was elevated in TNBC tissues and cells. High *SIPL1* expression was correlated with tumor progression and low survival of patients with TNBC. In TNBC cells, SIPL1 contributes to increased cell proliferation, apoptosis resistance, and migration by activating the PI3K/AKT and NF-κB signal pathways. Notably, we confirmed that *SIPL1* was transcriptionally regulated by MAZ.

## Data Availability Statement

The original contributions presented in the study are included in the article/**Supplementary Material**. Further inquiries can be directed to the corresponding author.

## Ethics Statement

The studies involving human participants were reviewed and approved by the Institutional Research Ethics Committee of the First Affiliated Hospital of Zhengzhou University. The patients/participants provided their written informed consent to participate in this study. The animal study was reviewed and approved by the Committee of the Ethics of Animal Experiments of Zhengzhou University.

## Author Contributions 

Conception and design: SZ, JW, and JH. Acquisition of data: SZ, JW, TL, SL, and JH. Analysis and interpretation of data: KC, SZ, and JH. Writing, review, and/or revision of the manuscript: SZ, JW, and JH. Study supervision: SZ and JH. All authors contributed to the article and approved the submitted version.

## Funding

This study was supported by medical science and technology project of Henan Province (172102310027).

## Conflict of Interest

The authors declare that the research was conducted in the absence of any commercial or financial relationships that could be construed as a potential conflict of interest.

## Publisher’s Note

All claims expressed in this article are solely those of the authors and do not necessarily represent those of their affiliated organizations, or those of the publisher, the editors and the reviewers. Any product that may be evaluated in this article, or claim that may be made by its manufacturer, is not guaranteed or endorsed by the publisher.
