# Supplementary material for: SIPL1, Regulated by MAZ, Promotes Tumor Progression and Predicts Poor Survival in Human Triple-Negative Breast Cancer
Source: Front Oncol. 2021 Dec 17;11:766790. doi: 10.3389/fonc.2021.766790 (PMC8718759; doi:10.3389/fonc.2021.766790)

**SIPL1, regulated by MAZ, promotes tumor progression and predicts poor survival in human triple-negative breast cancer**

He Juanjuan^1^, Wang Jing^1^, Li Teng^2^, Chen Kunlun^3^, Li Songchao^2^, Zhang Shaojin^2^

**Supplementary Table s1:** Primers for RT-qPCR

|  | Forward premier (5’-3’) | Reverse premier (5’-3’) |
| --- | --- | --- |
| SIPL1 | GCT ATT GCA GGTGGA GAC GA | GCC TCC TGA AGC TGAACA CT |
| GAPDH | GGTGAAGGTCGGAGTCAACGG | GAGGTCAATGAAGGGGTCATTG |
| MAZ | GGATCACCTCAACAGTCACGTC | GGCACTTTCTCCTCGTGTCGTA |
| **shRNA targeting sequence** | | |
| SIPL1#1 | GAGTCAGTTTCCTACACCA | |
| SIPL1# 2 | CCACCGUGGAAGGACAGAATT | |
| shNC | GATTCAGTTGACTACAACA | |
| shMAZ | CCUCAACAGUCACGUCAGATT | |

**Supplementary Table S2.** Clinicopathologic characteristics of 119 patients with TNBC

| **Clinical and pathologic Indexes** | **N =119** | **%** |
| --- | --- | --- |
| **Age, yr**  Mean ± SD  Range | 52  28-81 |  |
| **Menopause status**  Premenopausal  Postmenopausal | 65  54 | 55  45 |
| **T stage**  pT1  pT2  pT3  pT4 | | 45 | | --- | | 37 | | 26 | | 11 | | 38  31  22  9 |
| **Node involvement**  N0  N1  N2 | | 70 | | --- | | 33 | | 16 | | 59  28  13 |
| **Tumor grade**  G1  G2  G3 | | 65 | | --- | | 32 | | 22 | | 55  27  18 |
| **Histo**  Dustal  Others | | 94 | | --- | | 25 | | 79  21 |
| **Chemotherapy**  Adjuvant  No Chemotherapy | | 56 | | --- | | 63 | | 47  53 |
| **Adjuvant radiotherapy**  Yes  No | | 31 | | --- | | 88 | | 26  74 |

**Supple.fig.1**

A, RT-qPCR and western blotting assay analysis of SIPL1 expression in BT-549 cells with SIPL1-silencing and MAZ overexpression. B, RT-qPCR and western blotting assay analysis of SIPL1 expression in BT-549 cells with MAZ-silencing and SIPL1 overexpression. Expression levels were normalized by GAPDH expression. **P < 0.01; ***P < 0.001.


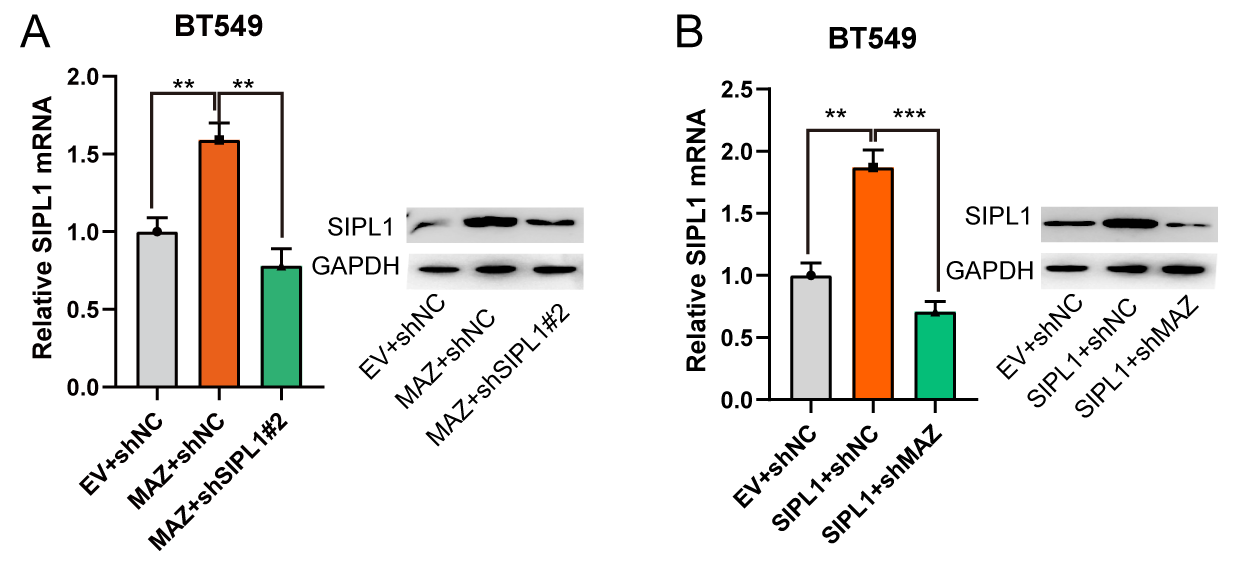

Supplement: Supplementary file 1 [file DataSheet_1.doc]
